# A SUMOylation/immune-related gene signature predicts the prognosis and immunotherapy efficacy of patients with triple-negative breast cancer

**DOI:** 10.7717/peerj.21139

**Published:** 2026-04-15

**Authors:** Pengcheng Zou, Hongyan Xu, Yang Gao, Weichao Bai, Xinhan Zhao, Shan Shao

**Affiliations:** Department of Oncology, The First Affiliated Hospital of Xi’an Jiaotong University, Xi’an, Shaanxi, China

**Keywords:** SUMOylation, Immune, Prognosis, Triple-negative breast cancer, Tumor microenvironment, Immunotherapy

## Abstract

**Background:**

SUMOylation, a type of posttranslational protein modification, is linked to numerous biological processes, including tumorigenesis, the immunological response, and DNA repair. Immune-related genes are crucial for immune monitoring and the formation of the tumor immunological microenvironment. The aim of this work was to create a survival prediction model that combines SUMOylation and immune-related gene expression to guide personalized treatment strategies for triple-negative breast cancer (TNBC) patients.

**Methods:**

RNA sequencing (RNA-seq) data and clinical information from TNBC patients were obtained from The Cancer Genome Atlas (TCGA) and the Gene Expression Omnibus (GEO). An unsupervised clustering method was applied to identify different SUMOylation-related subclusters in TNBC. Using immune cell infiltration levels and immunological scores, we performed weighted gene coexpression network analysis (WGCNA) to identify SUMOylated and immune-related genes (SIRGs). Three machine learning methods, namely, least absolute shrinkage and selection operator (LASSO), support vector machine recursive feature elimination (SVM-RFE), and random forest (RF), were utilized to construct a risk prognosis model from the SIRGs. The model genes were also analyzed through single-cell sequencing. Furthermore, we explored the correlations between risk score and patient prognosis, immune cell infiltration, and immunotherapy. The expression of characteristic genes was ultimately validated by quantitative real-time polymerase chain reaction (qRT-PCR) and immunohistochemistry (IHC).

**Results:**

The expression levels of genes (MITD1, IL12B, and ZP1) identified by machine learning were used to develop a prognostic model and nomogram. The low-risk patients had significantly better survival rates than the high-risk patients in both the training and validation cohorts. Specifically, patients in the low-risk group had increased immune cell infiltration, immune checkpoint gene expression, and sensitivity to immunotherapy.

**Conclusion:**

The constructed SIRG-related prognostic model can accurately predict prognosis and treatment efficacy for patients with TNBC.

## Introduction

Breast cancer (BRCA) is the most prevalent malignant cancer among women worldwide and has a high mortality rate. GLOBOCAN 2022 reported approximately 2.3 million new cases of BRCA and 665,684 deaths in 2022 (Bray et al., 2024). Triple-negative breast cancer (TNBC) is a special subtype of BRCA that accounts for 15–20% of all BRCA cases (Carey et al., 2010). Because it does not express specific molecules, including estrogen receptor, progesterone receptor, and human epidermal growth factor receptor 2, TNBC is resistant to endocrine and targeted therapy. Chemotherapy and surgical intervention are the primary treatments for TNBC (Leon-Ferre & Goetz, 2023). However, compared with other BRCA subtypes, TNBC is more heterogeneous and invasive, with most patients ultimately suffering from tumor recurrence and metastasis.

In recent years, immunotherapy has played an important role in antitumor treatment (Meric-Bernstam et al., 2021). Among BRCA patients, those with TNBC have derived the most significant advantage from immunotherapy (Liu et al., 2023). Compared with other subtypes, TNBC has a greater prevalence of tumor-infiltrating lymphocytes (TILs) and PD-L1 expression and is categorized as an “immune-enriched” or “hot” tumor (Leon-Ferre et al., 2024). Classic findings from the KEYNOTE-522, KEYNOTE-355, and IMpassion130 studies suggested that both early-stage and advanced-stage TNBC patients can benefit from immunotherapy (Schmid et al., 2018, 2020; Cortes et al., 2022). Nonetheless, only a fraction of TNBC patients receive clinical advantages from anti-PD-1/PD-L1 therapy. Given the heterogeneity of TNBC, identifying appropriate subsets of TNBC patients who can actually benefit from immunotherapy is essential.

Small ubiquitin-like modification (SUMO), or SUMOylation, is a posttranslational modification that involves the covalent attachment of SUMO proteins to a substrate protein through a sequence of enzymatic events (Matunis, Coutavas & Blobel, 1996). SUMOylation regulates biological processes, including cell proliferation, apoptosis, DNA damage repair, transcription, protein transport, and signal transduction (Hickey, Wilson & Hochstrasser, 2012). Notably, there is a connection between SUMOylation and immunity. The expression of immune checkpoint molecules such as PD-L1 and CTLA-4 can be modulated by SUMOylation (Ma et al., 2023; Wang et al., 2024). SUMOylation additionally influences the differentiation of immune cells including T cells and natural killer cells. SUMOylation can regulate T lymphocyte proliferation and differentiation, influencing their capacity to kill tumor cells (Garaude et al., 2008; Xiong et al., 2019). Furthermore, tumor cells can use SUMOylation to alter the expression of self-antigens and diminish immunogenicity, allowing the cells to evade immune surveillance (Demel et al., 2022). In general, SUMOylation contribute significantly to immune function. However, despite the acknowledged importance of SUMOylation, its function in TNBC remains inadequately studied and deserves further research.

In this study, we aimed to explore the significance of SUMOylation and immune-related genes (SIRGs) for the prognosis and immunotherapy response of TNBC patients *via* bioinformatics methods, with the goal of developing a prognostic model to facilitate more effective and individualized therapies for TNBC patients.

## Materials and Methods

### Data source and preprocessing

RNA-seq gene expression data in count format from 1,118 BRCA samples (133 TNBC samples) and 113 normal samples, along with clinical data, were downloaded from The Cancer Genome Atlas (TCGA) database (https://portal.gdc.cancer.gov/). The expression matrix was subsequently converted to transcripts per million (TPM) and normalized *via* log2(TPM+1) transformation. We also obtained RNA-seq data and clinical information from the Gene Expression Omnibus (GEO) database (https://www.ncbi.nlm.nih.gov/geo/, ID: GSE58812, GSE20711). GSE58812 and GSE20711 contain RNA-seq and clinical data from 107 and 27 TNBC patients, respectively. The mRNA expression data of the GSE58812 dataset were adjusted using log2(x+1) transformation to achieve uniformity. We integrated the datasets of the TCGA-TNBC and GEO-TNBC cohorts and applied the “ComBat” R package to eliminate batch effects across the disparate datasets (Leek et al., 2012). Samples with missing values were excluded, and ultimately, the data for 248 TNBC patients were retained for further study. We used the “caret” R package to randomly divide the merged TNBC patients into a training set and a test set at a 6:4 ratio (Kuhn, 2008).

### Construction of SUMOylation-related clusters

The keyword “SUMOylation” was used to search for relevant gene sets in the Molecular Signature Database (MSigDB, http://www.gsea-msigdb.org/gsea/msigdb/). A total of 169 genes related to SUMOylation were obtained (Table S1). To identify different SUMOylation expression patterns in TNBC, we performed unsupervised clustering of the samples in the training set according to their expression of SUMOylation-related genes. Consensus clustering was performed using the R package “ConsensusClusterPlus” and repeated 1,000 times to ensure the stability of the clustering results (Wilkerson & Hayes, 2010). We determined the optimal cluster number according to the smoothness and tendency of the cumulative distribution function (CDF) curve. We subsequently used the “survival” and “survminer” R packages to perform Kaplan‒Meier (KM) analysis on different SUMOylation clusters, clarifying the difference in survival between the two clusters. The “limma” R package was used to identify SUMOylation-related differentially expressed genes (SRDEGs) (Ritchie et al., 2015). SRDEGs with *P* values < 0.05 and absolute Log2FC values >0.585 were considered significant and screened for further analysis. Gene set variation analysis (GSVA) was performed using the R package “GSVA” to elucidate the pathway differences between different SUMOylation clusters (Hänzelmann, Castelo & Guinney, 2013).

### Identification of genes associated with SUMOylation and immunity

The SRDEG expression data were analyzed by WGCNA using the “WGCNA” R package to identify coexpression modules (Langfelder & Horvath, 2008). First, the ESTIMATE algorithm and single-sample gene set enrichment analysis (ssGSEA) were performed on all the samples in the training set to obtain immune scores and immune cell infiltration scores, including ImmuneScore and aDC, B cell, CD8^+^ T cell, DC, iDC, macrophage, mast cell, neutrophil, NK cell, pDC, T helper cell, Tfh, Th1 cell, Th2 cell, TIL, and Treg scores (Rooney et al., 2015; Charoentong et al., 2017). On the basis of the above results, WGCNA was used to screen the most immune-related genes among the SRDEGs. The “PickSoftThreshold” function was used to automatically select the soft threshold, and modules with different power values were subjected to scale-free and average connectivity analysis to obtain the topological overlap matrix (TOM) and corresponding dissimilarity matrix (1-TOM). Pearson correlation analysis was performed on the coexpression modules based on the ImmuneScore and ssGSEA score, and the modules with the highest correlation with the immune cell infiltration scores were selected as SUMOylation- and immunity-related genes (SIRGs). To validate the SIRGs screened by WGCNA, we used the R package “clusterProfiler” to perform Kyoto Encyclopedia of Genes and Genomes (KEGG) and Gene Ontology (GO) enrichment analyses to determine the biological functions of SIRGs (Yu et al., 2012; Wu et al., 2021).

### Establishment and validation of the SIRG prognostic model

Univariate Cox regression analysis was performed on the SIRGs to identify genes whose expression exhibited a statistically significant relationship with prognosis (*P* < 0.01). Three machine learning algorithms, namely, least absolute shrinkage and selection operator (LASSO), support vector machine recursive feature elimination (SVM-RFE) and random forest (RF), were used to identify key prognosis-related SIRGs. The LASSO algorithm was implemented using the “glmnet” R package, which uses tenfold cross validation to locate important genes accurately (Friedman, Hastie & Tibshirani, 2010; Engebretsen & Bohlin, 2019). For the RF algorithm, we used the “randomForest” R package to screen the main candidate genes (Paul et al., 2018). The best gene subset was identified according to the accuracy value determined using the SVM-RFE algorithm with the “e1071” R package (Sanz et al., 2018). The “ggvenn” R package was subsequently used to intersect the key genes identified by the three machine learning algorithms to obtain the optimal SUMOylation and immune-related genes (OSIRGs, the core genes for building the model) (Chen et al., 2021). A Cox proportional hazards model was constructed on the basis of OSIRGs to calculate the risk score for each patient, with regression coefficients estimated from the Cox proportional hazards regression model. Patients in the training set were divided into high-risk and low-risk groups on the basis of the median risk score. Kaplan–Meier analysis was performed on the high- and low-risk groups using the “survival” and “survminer” R packages, and receiver operating characteristic (ROC) analysis of survival at 2, 3, and 5 years was performed using the “timeROC” R package (Blanche, Dartigues & Jacqmin-Gadda, 2013). Finally, the prognostic model was validated in the previously randomly divided validation set.

### Construction of a nomogram model

We constructed a nomogram by integrating variables that have been identified as independent prognostic factors, including age, tumor stage, and the risk score (Iasonos et al., 2008). In the nomogram scoring system, the total score was obtained by adding all the variable scores for each sample. ROC curve analysis of 2-, 3-, and 5-year survival rates was performed using the “timeROC” R package. The calibration plot of the nomogram was used to describe the predictive value between the predicted 2-, 3-, and 5-year survival status and the actual observed results.

### Enrichment analysis related to pathways and functions

Gene set enrichment analysis (GSEA) and GSVA were conducted to analyze pathways and functions associated with the risk score (Subramanian et al., 2005). The GO enrichment analysis included all three levels: molecular function, cellular component, and biological process. In addition, we identified several pathways closely related to tumor occurrence and development from the literature, including estrogen, androgen, EGFR, hypoxia, JAK-STAT, MAPK, NF-kB, p53, PI3K, TGF-β, TNFa, Trail, VEGF, and WNT pathways (Schubert et al., 2018; Holland, Szalai & Saez-Rodriguez, 2020). We used GSVA to calculate the enrichment score of each pathway and evaluate the correlation between the risk score and the pathway enrichment score.

### Analysis of survival and immune infiltration of OSIRGs

The “survminer” and “survival” R packages were used to select the optimal survival-related cutoff value of OSIRG expression. We analyzed the difference in survival between patients with high and low expression of OSIRGs and evaluated whether the expression of OSIRGs differed between tumor and normal tissues. The “CIBERSORT” R package was used to evaluate the correlation between the expression level of OSIRGs and the level of infiltration of various immune cells (Chen et al., 2018).

### Exploration of the tumor immune microenvironment

To further explore the different levels of immune cell infiltration in the tumor microenvironment (TME) between the high-risk and low-risk groups, we used various algorithms from TIMER2.0 (https://bio.tools/timer20), including TIMER, CIBERSORT, QUANTISEQ, MCPCOUNTER, XCELL, and EPIC. We also used the ssGSEA algorithm to evaluate the differences in immune cell infiltration and immune function between the high- and low-risk groups (Hänzelmann, Castelo & Guinney, 2013). In addition, we used the ESTIMATE algorithm to assess the relationships between tumor purity and risk scores, including the ESTIMATE score, immune score, and stromal score (Yoshihara et al., 2013).

### Prediction of the response to immunotherapy

To explore the tumor immune profiles in the two risk groups, we assessed the expression levels of common immune checkpoint genes in the two risk groups. Additionally, we utilized two algorithms, immunophenoscore (IPS) and tumor immune dysfunction and exclusion (TIDE), to analyze the immunogenicity and immunoreactivity of the tumors and further explored the performance of the risk model in predicting the response to immunotherapy (Charoentong et al., 2017; Jiang et al., 2018). The IPS values of the BRCA patients were downloaded from The Cancer Immunome Atlas (TCIA) (https://tcia.at/home). TIDE scores were calculated using an online tool (http://tide.dfci.harvard.edu/).

### Analysis of the effects of chemotherapeutic drugs

With reference to the Genomics of Drug Sensitivity in Cancer (GDSC) database, the “pRRophetic” R package was used to evaluate the sensitivity of high-risk and low-risk patients to different drugs (Geeleher, Cox & Huang, 2014). The half-maximal inhibitory concentration (IC50) reflects the degree of drug sensitivity.

### Tumor mutation landscape

To determine the somatic mutation patterns of high-risk and low-risk TNBC patients, the TCGA-BRCA mutation annotation format (MAF) file was obtained from the TCGA data portal (http://tcga-data.nci.nih.gov/tcga/). The “maftools” R package was used to calculate the TMB score of each patient (Mayakonda et al., 2018).

### Single-cell sequencing data collection and processing

Single-cell RNA sequencing (scRNA-seq) data from 8 TNBC samples were downloaded from the GSE161529 dataset in the GEO database. For quality control, the raw gene expression matrix was filtered using the “Seurat” R package and selected according to the following criteria: nFeature_RNA > 200, nFeature_RNA < 8,000, percent.mt < 15, and percent.rb < 15 (Butler et al., 2018). The filtered scRNA-seq data were then normalized and scaled. The dimensionality of the scRNA-seq data was reduced using the principal component analysis (PCA), and t-distributed stochastic neighbor embedding (t-SNE) projection was applied to cluster and visualize the results. The cells were annotated according to their expression of typical cell surface markers. Finally, we analyzed the expression levels of OSIRGs in different clusters.

### Cell culture

The triple-negative breast cancer cell line MDA-MB-231 and normal breast epithelial MCF-10A cell line were both purchased from Procell Life Science & Technology (Wuhan, China). MDA-MB-231 cells were cultured in DMEM medium containing 10% fetal bovine serum (FBS) and 1% penicillin-streptomycin (double antibiotic). MCF-10A cells were cultured in MCF-10A cell specific medium (Shanghai Jinyuan Biotechnology, Shanghai, China). Both Cell lines were grown in a humidified atmosphere at 37 °C with 5% CO_2_.

### Quantitative real-time PCR

Total RNA was extracted from cultured cells using Trizol reagent (New Cell & Molecular Biotech, Suzhou, China). The concentration and purity of RNA were determined by NanoDrop spectrophotometry. Complementary DNA (cDNA) was synthesized from 1 μg of total RNA using PrimeScript RT Master Mix (Takara Bio, Kusatsu, Shiga, Japan). Quantitative real-time PCR was then performed using SYBR Green Master Mix (Polymer Medicine, China) on a CF96 Real-Time PCR Detection System. The reaction conditions included an initial denaturation at 95 °C for 30 s, followed by 40 cycles of 95 °C for 5 s and 60 °C for 30 s. Relative gene expression levels were calculated using the 2^(−ΔΔCt)^ method, with β-actin as the internal control. Each group included three independent biological replicates. Table S2 shows the primer sequences for Quantitative real-time PCR analysis.

### Immunohistochemical (IHC) staining

The 10 TNBC tissue samples and adjacent samples were obtained from patients at the First Affiliated Hospital of Xi’an Jiaotong University. The study was conducted in accordance with the Declaration of Helsinki and approved by the Ethics Committee of the First Affiliated Hospital of Xi’an Jiaotong University (protocol code: XJTU1AF2025LSYY-675; date: August 20, 2025). The requirement for informed consent was waived by the Ethics Committee due to the retrospective nature of the study and the use of anonymized samples (protocol code: XJTU1AF2025LSYY-675; date: August 20, 2025). Anti-MITD1 (rabbit polyclonal, AWA67480; Abiowell, Changsha, Hunan, China), anti-IL12B (rabbit polyclonal, AWA44817; Abiowell, Changsha, Hunan, China) and anti-ZP1 (rabbit polyclonal, AWA46715; Abiowell, Changsha, Hunan, China) antibodies were used for immunohistochemical staining. Two pathologists independently and blindly evaluated the results of the immunohistochemical staining. Semiquantitative scoring of the staining intensity was performed. The staining intensity was categorized as 0 (negative), 1 (light yellow), 2 (brown yellow), or 3 (dark brown). According to the proportion of positively stained cells among the total number of cells, 0–25% was scored as 1; 26–50% was scored as 2; 51–75% was scored as 3; and >75% was scored as 4. The staining intensity and staining proportion scores were multiplied to obtain the final score.

### Statistical analysis

The bioinformatics analysis, statistical analysis, and machine learning in this study were performed using R software (version 4.2.1; R Core Team, 2022) or GraphPad Prism 10. The log-rank test, Wilcoxon test, Kruskal‒Wallis test, and Spearman test were applied for statistical analysis. A *P* value < 0.05 was considered statistically significant.

## Results

### Identification of SUMOylation-related clusters in TNBC

**Figure 1:**
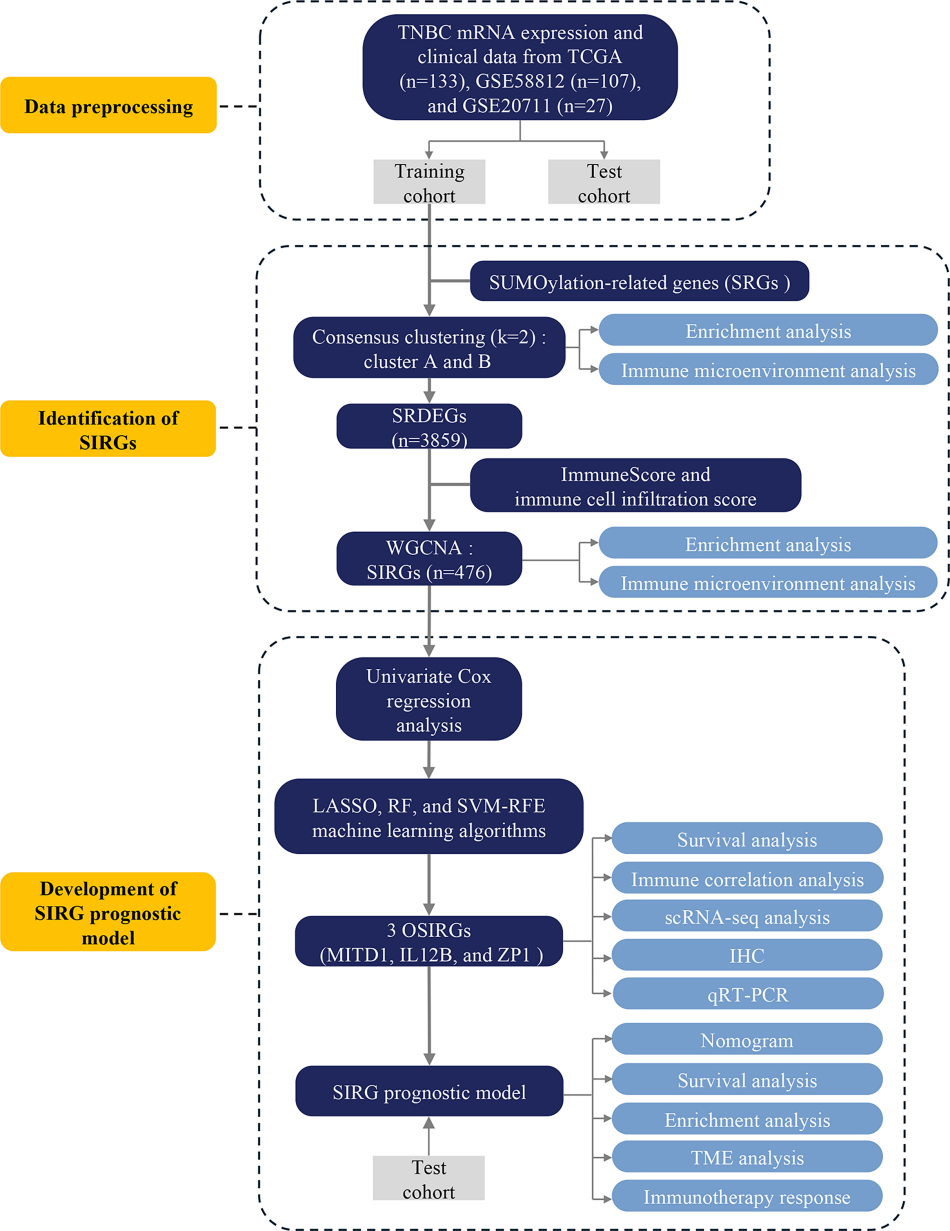
Flowchart of the study design.

**Figure 2:**
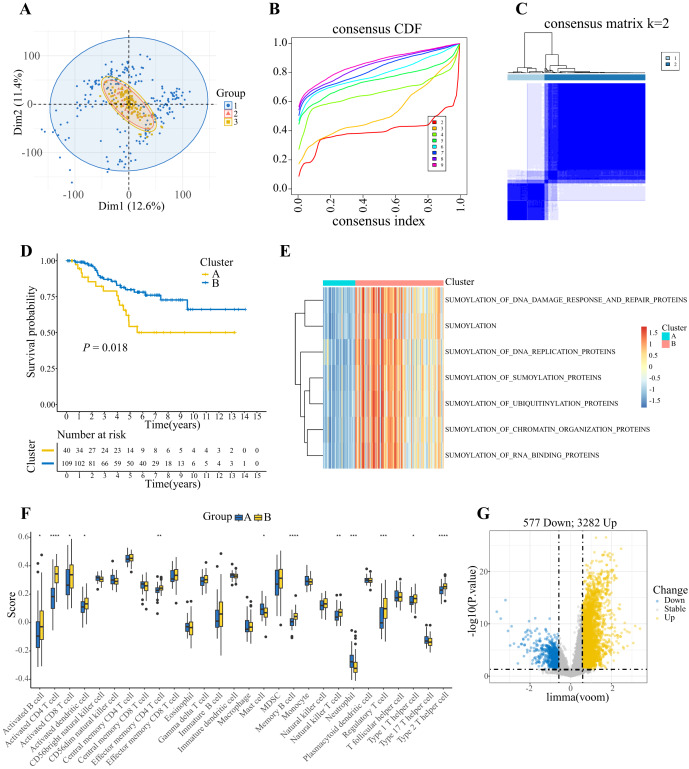
Process of identifying SUMOylation-related patient clusters. (A) Principal component analysis (PCA) of the shared gene expression profiles of the combined TCGA-TNBC and GEO-TNBC cohorts. (B) The relative change in the area under the cumulative distribution function (CDF) curve for k = 2–9. (C) Consensus clustering of TNBC patients for k = 2. (D) OS of patients in the two SUMOylation-related clusters (*P* = 0.018, log-rank test). (E) GSVA enrichment analysis showing the activation states of biological pathways in distinct SUMOylation clusters. (F) Abundance of TME cell infiltration between two clusters (*P < 0.05; **P < 0.01; ***P < 0.001; ****P < 0.0001). (G) 3,859 SRDEGs were screened according to the specific criteria applied (*P* < 0.05, |Log2FC| > 0.585).

The study workflow is shown in Fig. 1. Principal component analysis (PCA) revealed that the dispersion of the three datasets decreased after the samples were merged and batch effects were removed (Fig. 2A). We used an unsupervised clustering algorithm to divide the 149 TNBC patients in the training set into k clusters (k = 2–9) according to their expression of 169 SUMOylation-related genes (SRGs). The intragroup correlation was the strongest and the intergroup correlation was the weakest when k = 2 (Figs. 2B, 2C). Therefore, we considered TNBC to cluster into two SUMOylation-related subclusters, with 40 cases in cluster A and 109 cases in cluster B. We performed Kaplan‒Meier (KM) survival analysis on these two subclusters, and the results revealed that the overall survival of patients in cluster A was poor (Fig. 2D). The activation of SUMOylation-related pathways in cluster B was greater than that in cluster A, indicating that SUMOylation was enhanced in cluster B (Fig. 2E). GSVA was used to explore the functions of the genes with differences in expression between the two clusters. The results revealed that cluster A was enriched mainly in pathways such as energy metabolism and lipid metabolism, whereas cluster B was enriched mainly in cell cycle regulation and DNA damage repair (Fig. S1). Furthermore, we applied the ssGSEA method to TNBC samples in the training set to characterize immune infiltration in different SUMOylation-related clusters. As shown in Fig. 2F, cluster B had a greater abundance of antitumor immune cells, including activated B cells, activated CD4+ T cells, activated CD8+ T cells, *etc*. Differential expression analysis was performed on the two SUMOylation-related clusters, and 3,859 SUMOylation-related differentially expressed genes (SRDEGs) were screened and visualized by volcano plots (Fig. 2G).

### Identification of SUMOylation/immune-related genes

**Figure 3:**
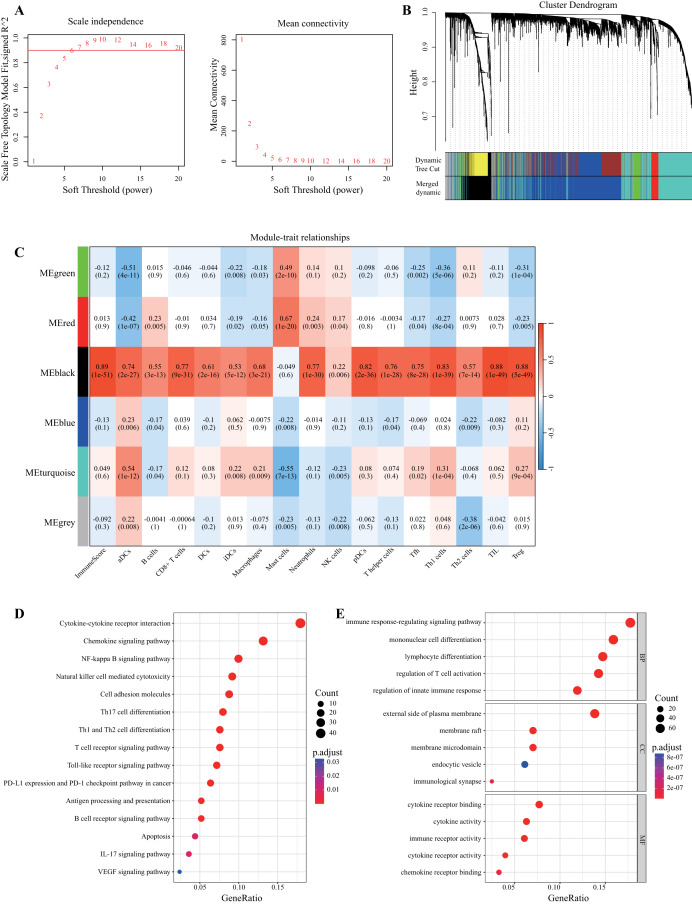
Discovery of prognostic SIRGs by WGCNA. (A) Trends of scale-free topology model fit and mean connectivity. (B) Gene modules identified by WGCNA. (C) Correlations between black modules and immune-related scores. (D, E) KEGG and GO enrichment analyses of SIRGs.

We applied the ESTIMATE algorithm and performed single-sample gene set enrichment analysis (ssGSEA) on all TNBC samples in the training set to obtain the immune score and immune cell infiltration score, respectively. SRDEGs were extracted to construct coexpression networks and modules. To identify the gene modules in SRDEG with the greatest relevance to immune infiltration, we performed WGCNA based on the immune score and immune cell infiltration score of the training set. The “pickSoftThreshold” function in the “WGCNA” R package automatically selected a soft threshold of 6 (Fig. 3A). Multiple gene modules were divided by the dynamic cutting method, and all modules were subsequently clustered using the “mergeCloseModules” function to obtain the final module (Fig. 3B). Using Pearson correlation analysis, the “black” module was selected as the module with the highest correlation with the immune scores (Fig. 3C). This module contained a total of 476 SUMOylated and immune-related genes (SIRGs). KEGG and GO enrichment analyses were used to validate the screened SIRGs. The KEGG results revealed that the genes in the “black” module were enriched mainly in the pathways of immune regulation, the inflammatory response, and cell signaling (Fig. 3D). The GO results revealed positive regulation of the immune response and cytokine activity (Fig. 3E).

### Development and validation of the SIRG prognostic model

**Figure 4:**
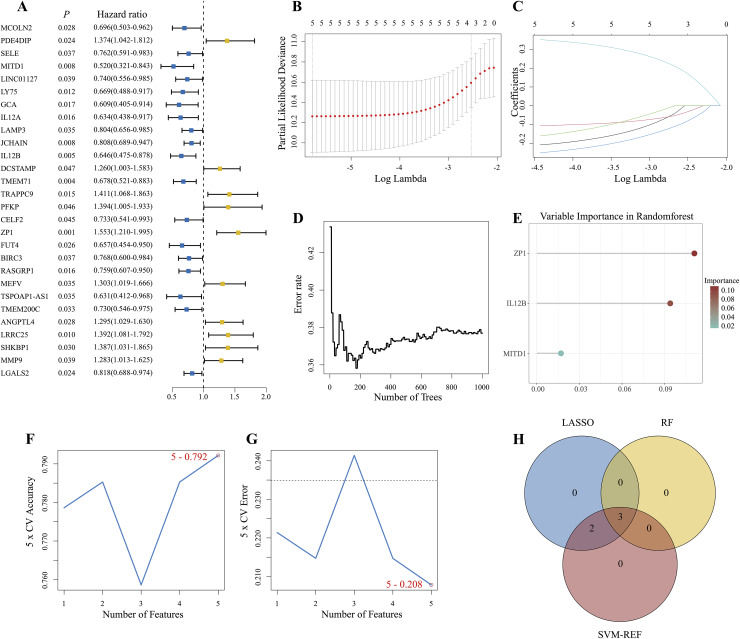
Machine learning analysis to identify prognostic signatures. (A) Forest plot of univariate Cox regression analysis. (B, C) The best value of lambda was selected through LASSO regression. (D, E) Error graphs and importance indices of the RF models. (F, G) The SVM-RFE algorithm was used to screen potential key genes. The X-axis represents the λ value, and the Y-axis represents the cross-validation accuracy and error. (H) Overlapping genes among genes screened by LASSO regression, random forest and the SVM-RFE algorithm.

Through univariate Cox analysis, we identified 28 genes that were significantly associated with survival (Fig. 4A). To identify the key signature genes, five candidate genes with *P* < 0.001 were submitted to the LASSO, RF, and SVM-RFE algorithms. Five gene signatures were identified by LASSO with 10-fold cross validation (Figs. 4B, 4C). Important genes were identified computationally using the RF algorithm (Figs. 4D, 4E). Five genes were screened using the SVM-RFE algorithm, with a 10-fold cross-validation accuracy of 0.792 (Figs. 4F, 4G). To obtain the optimal SUMOylation genes and immune-related genes (OSIRGs), we intersected the genes screened by the above three algorithms (Fig. 4H), which yielded three robust signature genes: MITD1, IL12B, and ZP1. A Cox proportional hazards model was subsequently constructed on the basis of the expression levels of these three genes. The final risk score was calculated as follows: risk score = (−0.4201113 * MITD1 exp.) + (−0.2983738 * IL12B exp.) + (0.3439366 * ZP1 exp.).

**Figure 5:**
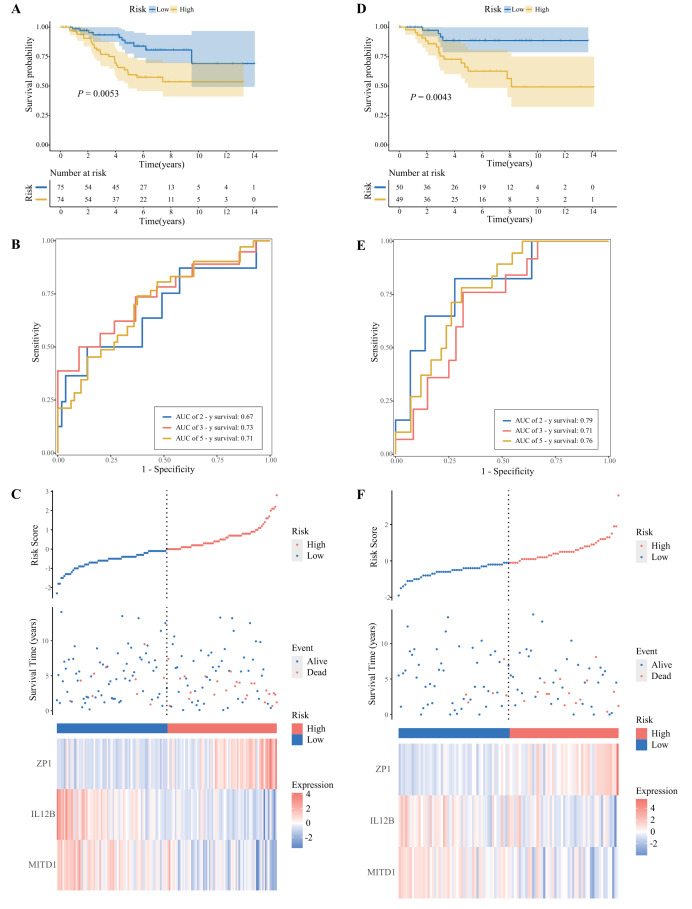
Development and validation of the SIRG prognostic model. (A, D) OS of the high- and low-risk groups in the training and test cohorts (log-rank test). (B, E) ROC curves for predictive efficiency in the training and test cohorts (blue, 2 years; orange, 3 years; yellow, 5 years). (C, F) Ranked dots of the risk score, scatter plots of patient survival status and heatmaps of the prognostic signature in the training and test cohorts.

To explore the characteristics of patients with different risk scores, we divided the training cohort into a high-risk group and a low-risk group according to the median risk score. The survival curve (Fig. 5A) revealed a significant difference in survival time between the high-risk group and the low-risk group within the training set (*P* = 0.0053). The overall survival (OS) of high-risk patients was significantly lower than that of low-risk patients. In addition, we performed a time-dependent receiver operating characteristic (ROC) analysis (Fig. 5B) to calculate the area under the curve (AUC) value of the prognostic model; the AUC values for 2 years, 3 years, and 5 years were 0.67, 0.73, and 0.71, respectively. Figure 5C shows the distribution of risk scores and OSIRG expression between the two risk groups.

To verify the reliability of the prognostic model, we then calculated the risk score of each patient in the validation set and divided the validation cohort into a high-risk group and a low-risk group according to the median risk score. KM analysis suggested that the OS of patients in the high-risk group was also significantly lower than that of patients in the low-risk group (Fig. 5D). The ROC curve performed well (Fig. 5E), with AUC values of 0.79, 0.71, and 0.76 for 2, 3, and 5 years, respectively. These findings indicate that the model has good predictive ability for the long-term prognosis of breast cancer patients in the validation set. Additionally, Fig. 5F shows the distributions of risk scores and model gene expression in the two risk groups. Therefore, the training and validation sets show similar trends, indicating that the SIRG prognostic model is stable and reliable.

**Figure 6:**
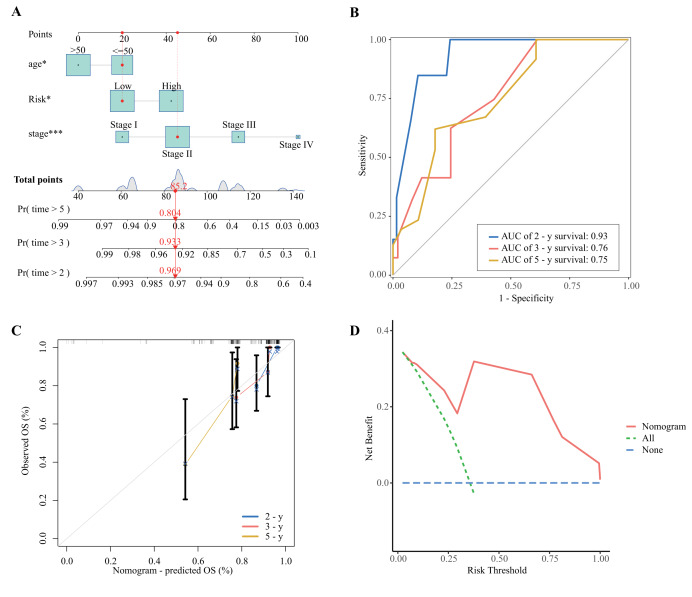
Construction and validation of a nomogram model. (A) Nomograms for 2-, 3-, and 5-year overall survival prediction. The red line shows an example of how to determine the prognosis (*P < 0.05; ***P < 0.001). (B) 2-, 3-, and 5-year OS ROC curves for the nomogram. (C, D) Calibration plots of the nomogram.

The clinical characteristics of the high- and low-risk groups are shown in Fig. S2A. The Sankey diagram depicts the distribution of TNBC patients in terms of clinical stage, high- and low-risk status, and survival status (Fig. S2B). Cox multivariate analysis revealed that age, stage, and the risk score were independent prognostic factors. Thus, using these independent prognostic factors, we developed a nomogram to predict the 2-, 3-, and 5-year survival rates of breast cancer patients (Fig. 6A). The AUC values at 2, 3, and 5 years were 0.93, 0.76, and 0.76, respectively, indicating that the nomogram had good predictive performance (Fig. 6B). The calibration curves revealed a high degree of consistency between the predicted OS and the actual OS (Figs. 6C, 6D).

### Analysis of OSIRGs

**Figure 7:**
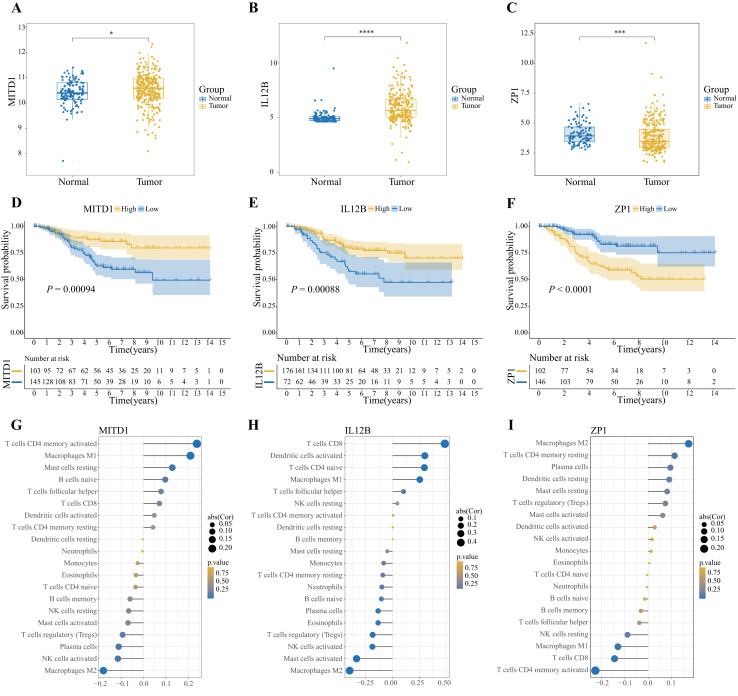
Exploration of OSIRGS. (A–C) KM curves of OS for high- and low-risk patients in the training and validation sets (*P < 0.05; ***P < 0.001; ****P < 0.0001). (D–F) Changes in the expression of OSIRs between normal and tumor tissues. (G–I) Associations between OSIRs and immune infiltration levels. The color represents the significance level. The circle size represents the correlation coefficient.

Further analysis of the genes used to construct the model revealed that the expression of MITD1 and IL12B in TNBC tissues was greater than that in normal tissues, whereas the expression of ZP1 exhibited the opposite pattern (Figs. 7A–7C). KM analysis revealed that when ZP1 was highly expressed, patient survival time was shorter. When IL12B and MITD1 were highly expressed, patient prognosis improved (Figs. 7D–7F). In terms of immune cell infiltration, the expression levels of IL12B and MITD1 were the most negatively correlated with the infiltration level of M2 macrophages (R = −0.41, *P* < 0.001; R = −0.18, *P* = 0.0046) (Figs. 7G, 7H). In contrast, ZP1 had the strongest positive correlation with the infiltration level of M2 macrophages (R = 0.14, *P* = 0.024) (Fig. 7I). In addition, IL12B was strongly correlated with CD8^+^ T cells (R = 0.54, *P* < 0.001).

### Enrichment analysis associated with risk subtypes

**Figure 8:**
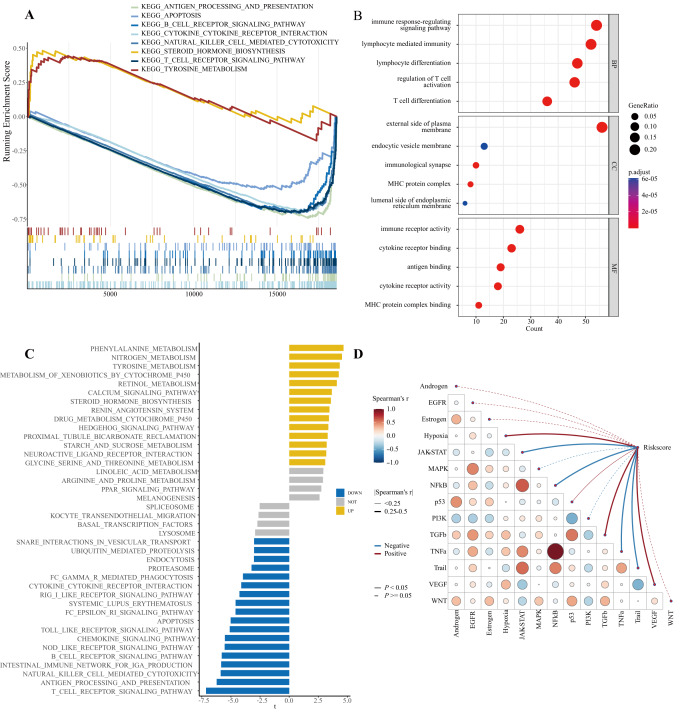
Biological functions. (A) Significantly enriched pathways in the high- and low-risk groups. The extremum located on the left indicates a positive association between risk scores and pathway activity, and vice versa. (B) Bar plot for GO enrichment, with bar length representing the degree of enrichment and color representing the degree of difference. (C) Significant differences in pathways between the high- and low-risk groups. (D) Correlations between the risk core and important pathways in tumors.

GSEA (Fig. 8A) revealed that tyrosine metabolism and steroid hormone biosynthesis were active in the high-risk group, whereas apoptosis, cytokine‒cytokine receptor interaction, antigen processing and presentation, natural killer cell-mediated cytotoxicity, T-cell receptor signaling, and B-cell receptor signaling were the pathways active in the low-risk group. GO functional analysis (Fig. 8B) identified mainly immune response terms, such as immune response regulation signaling pathway, lymphocyte-mediated immunity, and lymphocyte differentiation. GSVA enrichment analysis results (Fig. 8C) showed that the high-risk group was enriched mainly in cell function and metabolism pathways, such as endocytosis, proteasome, lysosome, phenylalanine metabolism, nitrogen metabolism, tyrosine metabolism, and steroid hormone biosynthesis. The low-risk group was enriched mainly in immune-related pathways, such as T cell receptor signaling, antigen processing and presentation, natural killer cell-mediated cytotoxicity, IgA production in the intestinal immune network, B-cell receptor signaling, NOD-like receptor signaling, chemokine signaling, Toll-like receptor signaling, and apoptosis pathways. In addition, the hypoxia, JAK-STAT, NF-kB, p53, TGFb, TNFa, Trail, and VEGF pathways were associated with the risk score (Fig. 8D).

### Characteristics of the tumor microenvironment and mutation landscape in different risk subtypes

**Figure 9:**
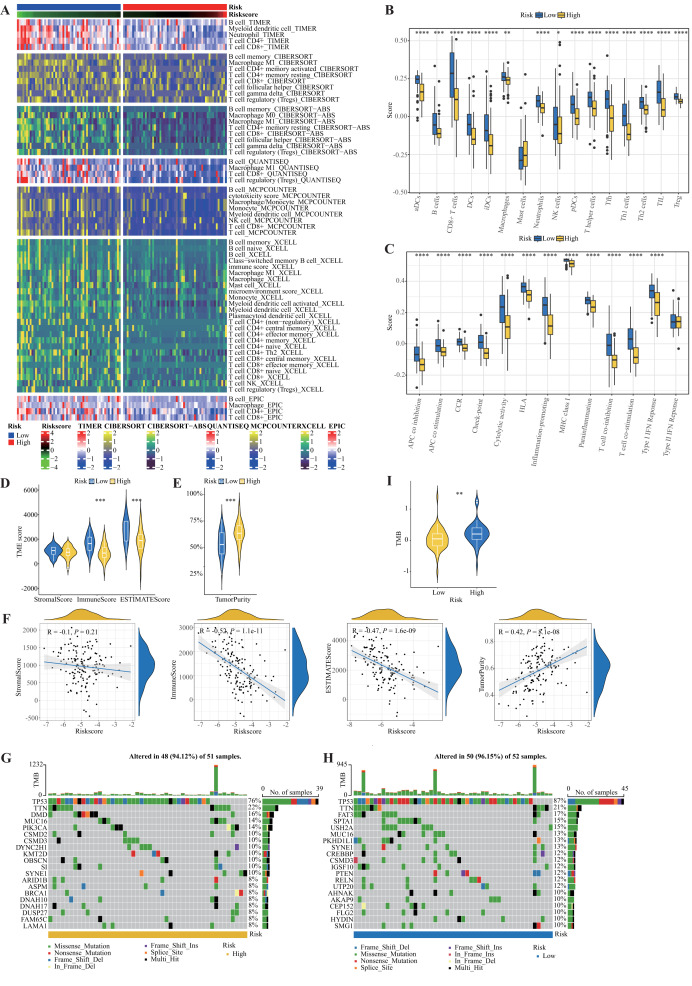
Immune-related analysis. (A) Relationships between the risk score and tumor immune infiltration according to evidence from the TIMER database. (B, C) Abundances of 16 immune cell types and immune pathway scores in the risk groups according to ssGSEA. Asterisks indicate significance (*P < 0.05; **P < 0.01; ***P < 0.001; ****P < 0.0001). (D–F) Correlation between the TME score and risk score. (G–I) Landscape of TMB and tumor mutation status in the high- and low-risk groups.

TIMER database analysis revealed differences in the abundance of infiltrating immune cells between the high-risk and low-risk groups (Fig. 9A). The infiltration of CD4+ T cells, CD8+ T cells, and B cells in the low-risk group was significantly greater than that in the high-risk group. The results of the ssGSEA were divided into two parts: immune cells and immune function (Figs. 9B, 9C). In the low-risk group, except for mast cells, most immune cells exhibited highly abundant infiltration, and APC coinhibition, APC costimulation, chemokine receptor, checkpoint, cytolytic, HLA, proinflammatory, MHC class I molecule, parainflammation, T-cell coinhibition, T-cell costimulation, and type I IFN response responses were significantly greater in the low-risk group than in the high-risk group. To further analyze the relationship between the TME and the risk score, we used the “estimate” R package to calculate TME scores (StromalScore, ImmuneScore, ESTIMATEScore, and TumorPurity) for each patient (Figs. 9D–9F). The results revealed significant differences in ImmuneScore, ESTIMATEScore, and TumorPurity between the high-risk and low-risk groups. Among the different TME scores calculated, ESTIMATEScore was positively correlated with the risk score (R = 0.42, *P* < 0.0001). ImmuneScore and ESTIMATEScore were both statistically significantly negatively correlated with the risk score. We subsequently studied the genomic characteristics of different risk subgroups in the TCGA-TNBC dataset. The waterfall plot and violin plot revealed that the TMB in the low-risk group was greater than that in the high-risk group (Figs. 9G–9I).

### Prediction of immunotherapy response and drug sensitivity

**Figure 10:**
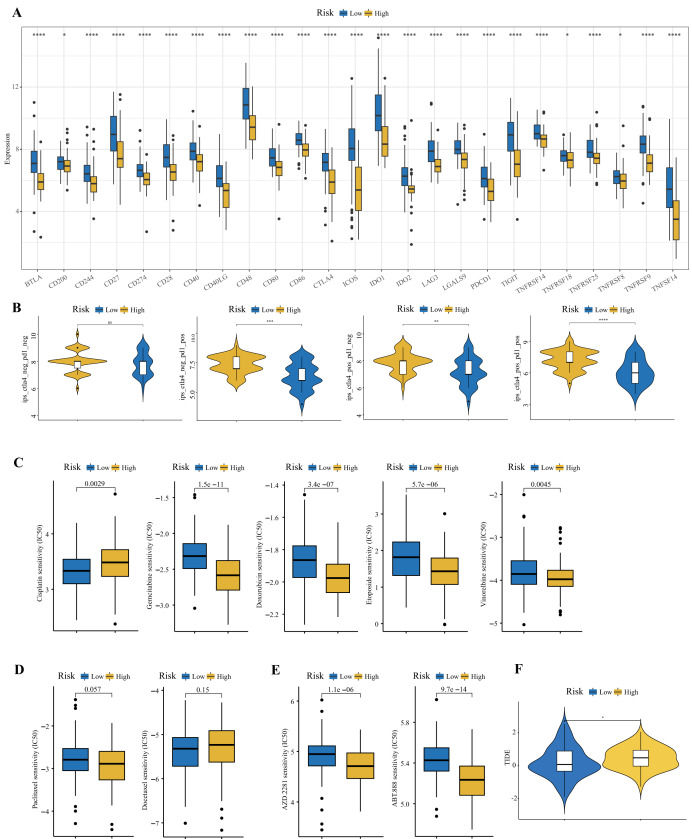
Prediction of immunotherapy response in the high- and low-risk groups. (A) Differences in the expression levels of immune checkpoints between the high- and low-risk groups. (B) Comparisons of IPS between the two risk groups. (C–E) Relationships between the risk score and chemotherapeutic sensitivity (Wilcoxon test). (F) Differences in TIDE score between the two risk groups (ns, not significant; *P < 0.05; **P < 0.01; ***P < 0.001; ****P < 0.0001).

We observed that multiple immune checkpoint genes, including PD1, PDL1, and CTLA4, were highly expressed in the low-risk group (Fig. 10A). To further explore the implications of this observation, we calculated the immunophenotypic score (IPS) for each patient using the TCIA database to evaluate the immunotherapy responses of TNBC patients in the high- and low-risk groups. Patients were stratified according to the levels of PD1 and CTLA4 expression. The results revealed that low-risk patients presented higher values in all strata, except in cases when patients were negative for both PD1 and CTLA4 (Fig. 10B). These findings strongly suggest that TNBC patients in the low-risk group respond better to ICI therapy. In addition, we performed tumor immune dysfunction and exclusion (TIDE) analysis on the low-risk group. The results revealed that patients in the low-risk group had lower TIDE scores (Fig. 10F). Lower TIDE scores are correlated with a lower likelihood of immune escape and thus greater efficacy of immunotherapy. Drug sensitivity analysis revealed that TNBC patients in the high-risk group were more sensitive to doxorubicin, etoposide, gemcitabine, vincristine, veliparib (ABT.888), and olaparib (AZD.2281), whereas patients in the low-risk group were more sensitive to cisplatin (Figs. 10C–10E).

### Characterization of OSIRGs by scRNA-seq analysis

**Figure 11:**
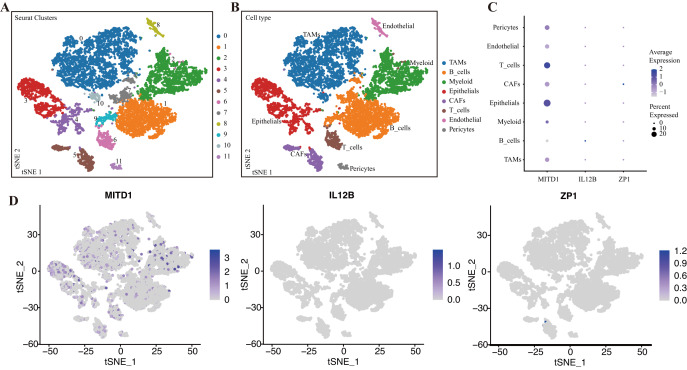
Verification of OSIRGs through scRNA-seq. (A, B) The t-SNE plot shows the results of the dimension reduction cluster analysis, and the cells were annotated as eight different cell types. (C, D) The expression of signature genes in TNBC visualized *via* t-SNE and bubble plots.

We downloaded the GSE161529 single-cell dataset of eight TNBC samples from GEO. By screening total cells according to intracellular gene features, chromosomal gene percentages, *etc*., a total of 10,811 cells were obtained, and PCA was subsequently used for latitude reduction. The top 14 PCs were retained for further t-SNE to obtain 13 cell subsets (Fig. 11A). Using canonical markers defined in the literature, the cells were divided into the following clusters: TAMs, B cells, myeloid cells, epithelial cells, endothelial cells, T cells, CAFs and pericytes (Fig. 11B). To study the expression of model genes in different cells, we visualized these data in t-SNE and bubble plots (Figs. 11C, 11D). The results showed that MITD1 was highly expressed in epithelial and T cells.

### Validation of OSIRG expression

**Figure 12:**
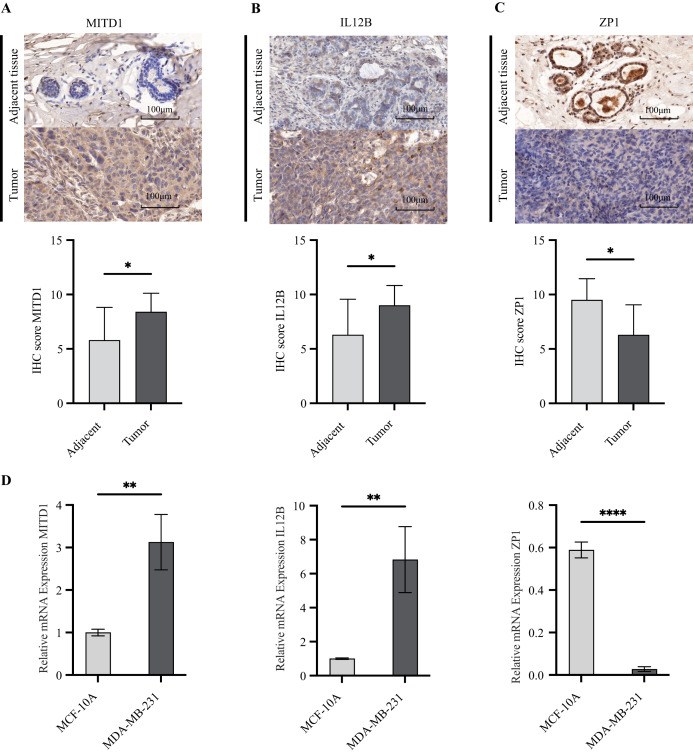
Expression of OSIRGs in TNBC and adjacent tissues. (A–C) IHC analysis of MITD1, IL12B and ZP1 expression (*P < 0.05, **P < 0.01, ****P < 0.0001). (D) Pcr verified the expression levels of OSIRGs in normal and tumor cells.

To explore the differences in the protein expression of OSIRGs in tumor and adjacent tissues, we used immunohistochemical staining to detect the expression levels of MITD1, IL12B, and ZP1 in tissue samples. The results revealed that the expression of MITD1 and IL12B in TNBC tissues was greater than that in adjacent tissues (Figs. 12A, 12B), whereas ZP1 expression exhibited the opposite pattern (Fig. 12C). qRT-PCR results showed that the expression levels of MITD1 and IL-12B were significantly higher in MDA-MB-231 cells, while ZP1 was the opposite (Fig. 12D).

## Discussion

In recent years, improvements in immunotherapy have been of great importance for TNBC treatment. Compared with other subtypes of breast cancer, TNBC has unique molecular characteristics, including high expression of PD-L1, a high tumor mutation load, and more tumor-infiltrating lymphocytes (TILs), and is more susceptible to immunotherapy. Some clinical studies, such as KEYNOTE-522 and KEYNOTE-355, have confirmed the clinical benefits of pembrolizumab combined with chemotherapy in patients with early and advanced TNBC. Although immunotherapy has improved the prognosis of many TNBC patients, only some patients benefit from this treatment. Therefore, finding biomarkers of genomic characteristics to predict and select patients who are most likely to benefit from immunotherapy will help achieve precision treatment.

The interactions between SUMOylation and tumorigenesis, metastasis and progression have been studied extensively. In TNBC, SENP1 overexpression promotes CSN5-mediated degradation of the ZEB1 protein through deSUMOylation of GATA1, ultimately enhancing tumor cell metastasis and invasion (Gao et al., 2022). Increasing evidence suggests that SUMO modification also plays an important role in the tumor immune microenvironment (TIME), especially in regulating immune cell function and immune escape mechanisms. For example, SENP3 deficiency-mediated AKT1 SUMOylation leads to AKT1 hyperphosphorylation and activation, promoting M2 polarization of macrophages (Gu et al., 2023). M2 macrophages release EGF, MMPs, VEGF and TGFβ, promoting tumor proliferation, invasion, angiogenesis and immune escape. In addition, SUMOylation affects T cell and B cell development, activation and function, thereby regulating the TME (Muromoto et al., 2006; Wang et al., 2015; Ding et al., 2016).

Although previous studies have explored the role of SUMOylation in breast cancer, most have focused on a limited number of SUMOylation-related proteins rather than delving into the comprehensive role of SUMOylation in TNBC or the interaction between TME cell infiltration and SUMOylation. We sought to address these gaps from a new perspective by evaluating the overlap of SUMOylation-related genes and immune-related genes and constructed a prognostic model through bioinformatics methods to evaluate the prognosis of TNBC patients and their response to immunotherapy.

In this work, we first divided the training set of TNBC patients into two subclusters, cluster A and cluster B, with different expression levels of SUMOylation-related genes that were defined using an unsupervised clustering algorithm. Compared with patients in cluster A, patients in cluster B had better OS and greater activation of SUMOylation-related pathways. TME analysis of the two clusters revealed that cluster B was enriched in many immune cell types, including activated CD8^+^ T cells, activated CD4^+^ T cells, B cells, and dendritic cells. CD8^+^ T cells and CD4^+^ T cells are key cells involved in antitumor immunity. Thus, their stronger infiltration may partially explain the better prognosis of patients in cluster B. Many studies have shown that SUMOylation plays an important role in regulating T-cell-related activities. Xiong et al. (2019) reported that UBC9 can mediate SUMOylation of the TCR adaptor protein SLP-76, increase IL-2 transcription, and thus regulate T-cell function. Another study revealed that PLC-γ1 SUMOylation is regulated by SUMO1 and PIASxβ/3 after TCR stimulation, which promotes the assembly of PLC-γ1 microclusters and is beneficial for T-cell activation (Wang et al., 2019).

Next, we identified immune-related genes from the SRGs *via* the WCGNA algorithm. Three OSIRGs (MITD1, IL12B, and ZP1) were finally identified through machine learning to construct the prognostic model. Dong et al. reported that MITD1 in breast cancer can inhibit tumor cell proliferation and migration, and immunohistochemistry demonstrated that the expression level of the MITD1 protein in breast cancer tissue was lower than that in normal tissue (Dong et al., 2022). Our study revealed a strong correlation between reduced MITD1 expression levels and poor prognosis in TNBC patients, consistent with the findings of previous studies. However, our immunohistochemistry results were opposite to those in the previous work, which may be related to differences in the types of tissue samples analyzed in the two studies or to the heterogeneity of breast cancer. Correlation analysis between MITD1 and immune cell infiltration revealed that the expression of MITD1 was positively correlated with the infiltration of B cells, CD4+ T cells, CD8+ T cells, dendritic cells, macrophages, and neutrophils. IL12B encodes the IL12 p40 subunit, which forms a heterodimer with the IL12 p35 subunit (IL12A) to form IL-12 and can also form a heterodimer with the IL23 p19 subunit (IL23A) to form IL-23. IL-12 and IL-23 are two important cytokines involved in cell-mediated immunity. Studies have shown that CD8^+^ T cells activated by IL-12 can induce apoptosis in breast cancer cells and inhibit tumor growth. Local injection of the IL-12 plasmid in TNBC can directly promote the expansion of CD8^+^ T cells and induce the expression of CXCR3-related genes, thereby enhancing the therapeutic effect of anti-PD-1 antibodies (Telli et al., 2021). Moreover, the balance between Th17 cells and Treg cells is a key factor in maintaining the normal immune response, and IL23 can promote the transformation of naive T cells into Th17 cells *in vivo* (Taherian et al., 2014; Lee, 2018). In this study, high expression of IL12B in TNBC was associated with a better prognosis. In addition, the expression of IL12B was positively correlated with the infiltration of antitumor immune cells (such as CD8^+^ T cells, NK cells and DCs), which is consistent with previous reports. ZP1 is known mainly for its association with the zona pellucida (ZP) of oocytes and the fertilization process. However, recent studies have shown that ZP1 may also play an important role in some tumors, and especially in the proliferation, migration and immune escape of tumor cells. ZP is composed of four glycoproteins, namely, ZP1, ZP2, ZP3 and ZP4. Studies have shown that ZP2 promotes the proliferation of colon cancer tumor cells through the ERK1/2-cyclin D1 signaling pathway (Kraus et al., 2021). In addition, ZP3 has been shown to promote tumor growth and regulate immunity in a variety of cancers, such as prostate cancer, pancreatic cancer, and lung adenocarcinoma (Costa et al., 2018; Pulawska‐Moon et al., 2024; Lyu & Li, 2025). Rahman et al. (2012) developed a new treatment method based on immune targeting of recombinant human ZP3, which was confirmed to be effective in a transgenic mouse model of ovarian granulosa cell carcinoma. However, research on ZP1 in tumors is still lacking. In this study, we confirmed the expression of the ZP1 protein in TNBC *via* immunohistochemistry and also demonstrated that high expression of ZP1 was associated with poor prognosis in TNBC patients. Immune infiltration analysis revealed that the expression level of ZP1 was positively correlated with the abundance of M2 macrophages and with Treg infiltration. Both M2 macrophages and Treg cells have immunosuppressive effects and are involved in immune escape, tumor metastasis, drug resistance, and other malignant behaviors. The specific biological mechanism of ZP1 activity in TNBC still needs further in-depth study.

In this study, we used the three OSIRGs mentioned above to develop a prognostic model based on data from the training cohort, which successfully distinguished TNBC patients into high-risk and low-risk groups. The effectiveness of the model was verified in the test cohort, and its diagnostic efficacy was confirmed by ROC curve analysis. In addition, the quantitative nomogram based on the risk score, age and tumor stage was helpful for the prognostic stratification of TNBC patients, further promoting the clinical application of the model. The enrichment analysis revealed that the genes in the low-risk group were related mainly to immune-related functions and pathways. In addition, we used the TIMER and ssGSEA algorithms to analyze immune infiltration in the high-risk and low-risk groups. The results revealed that most immune cells exhibited low infiltration abundance in the high-risk group. Although there was no significant difference in the stromal score between the high-risk group and the low-risk group, the high-risk group had a lower immune score. This finding also indirectly confirmed the poor prognosis of the high-risk group.

Immune checkpoints have been shown to be effective biomarkers for predicting TNBC response to ICI therapy (Xiao et al., 2019; Wang et al., 2023). Our analysis of immune checkpoint expression in the high-risk and low-risk groups of TNBC patients revealed that the expression levels of immune checkpoints, including CD274 (PD-L1), PDCD1 (PD-1), CTLA4, and BTLA, were increased in the low-risk group. In addition, the low-risk group had a greater tumor mutation burden, greater IPS score and lower TIDE score. In summary, our findings indicate that the low-risk group may be more sensitive to ICI treatment. In addition, we explored the relationship between risk score and chemotherapy efficacy. We analyzed commonly used chemotherapy drugs for TNBC, such as cisplatin, docetaxel, doxorubicin, etoposide, gemcitabine, paclitaxel, and vinorelbine. We found that the IC50 values of these drugs were different between the high-risk and low-risk groups. In addition, a recent study revealed that PARP inhibitors have significant efficacy in patients with BRCA mutations, providing a new direction for personalized treatment (Morganti et al., 2024). Therefore, we also explored the differences in the sensitivity of high-risk and low-risk groups to PARP inhibitors, and the results revealed that the high-risk group was more suitable for “ABT.888” (Veliparib) and “AZD.2281” (olaparib) treatment. In summary, the above results show that the TNBC risk score has a unique role in predicting the effects of immunotherapy and chemotherapy and in guiding clinical treatment.

scRNA-seq analysis revealed that MITD1 was highly expressed in various cells in the tumor microenvironment, including T cells, epithelial cells, macrophages, and endothelial cells. Therefore, we speculate that MITD1 expression has important relationships with immune activation and the immune microenvironment.

Although our analysis of SUMOylation in TNBC was comprehensive, this study has several limitations. First, the SUMOylation-related genes we collected and used for analysis may not be complete, which may introduce some bias to our study. Moreover, potential biases inherent in public datasets such as TCGA, including demographic and clinical heterogeneity, should be acknowledged. These factors may influence the generalizability of the proposed model, and further validation in independent clinical cohorts is warranted. Second, the omics data that we analyzed included only mRNA levels, while the SUMOylation process depends on protein activity. In the future, we will incorporate proteomics research and conduct experimental studies to elucidate their SUMOylation status and functional roles. Third, our study lacks validation in clinical datasets. Due to current limitations in data availability, such validation could not be performed in the present study. In addition, the number of TNBC samples in the publicly available single-cell RNA sequencing datasets used in this study was limited. Future studies incorporating larger, well-annotated TNBC scRNA-seq cohorts or harmonized multi-dataset integration with robust batch-correction methods will be necessary to achieve more refined cell-type resolution and deeper mechanistic insights. Finally, the specific mechanisms involved in the interactions between the model genes and immune cell infiltration in the TME still need further study.

## Conclusions

In summary, this study combined machine learning and bioinformatics methods to establish a prognostic model for TNBC based on three OSIRGs. With this model, the prognosis of TNBC patients can be accurately predicted, and the degree of immune infiltration and immunotherapy efficacy can be predicted, guiding clinicians to choose the best treatment strategy for each patient and achieve personalized treatment.

## Supplemental Information

10.7717/peerj.21139/supp-1 Supplemental Information 1 GSVA enrichment analysis showing the activation states of biological pathways in two SUMOylation-related clusters.

10.7717/peerj.21139/supp-2 Supplemental Information 2 Relationship between clinical characteristics and risk scores.

10.7717/peerj.21139/supp-3 Supplemental Information 3 The 169 SUMOylation related genes.

10.7717/peerj.21139/supp-4 Supplemental Information 4 MIQE checklist.

10.7717/peerj.21139/supp-5 Supplemental Information 5 Primers.

10.7717/peerj.21139/supp-6 Supplemental Information 6 Code.

10.7717/peerj.21139/supp-7 Supplemental Information 7 IHC original data.

10.7717/peerj.21139/supp-8 Supplemental Information 8 qRTPCR original data.

## Additional Information and Declarations

### Data Availability

The following information was supplied regarding data availability:

The code is available in the Supplemental File.

The Raw Data is available at Mendley and Baidu:

- Zou, Pengcheng (2025), “Raw Data”, Mendeley Data, V1, doi: https://doi.org/10.17632/mpkvr7wwkt.1.

- https://pan.baidu.com/s/10yWNkYYHx5bCLSXSTDYz0w?pwd=eajc.
