# Supplementary material for: A SUMOylation/immune-related gene signature predicts the prognosis and immunotherapy efficacy of patients with triple-negative breast cancer
Source: PeerJ. 2026 Apr 15;14:e21139. doi: 10.7717/peerj.21139 (PMC13091581; doi:10.7717/peerj.21139)
Supplement: Supplemental Information 1 [file peerj-14-21139-s001.docx]

**Supplementary Figure**

**Article Title**: A SUMOylation/immune-related Gene Signature Predicts the Prognosis and Immunotherapy Efficacy of Patients with Triple-Negative Breast Cancer.

**Fig. S1**


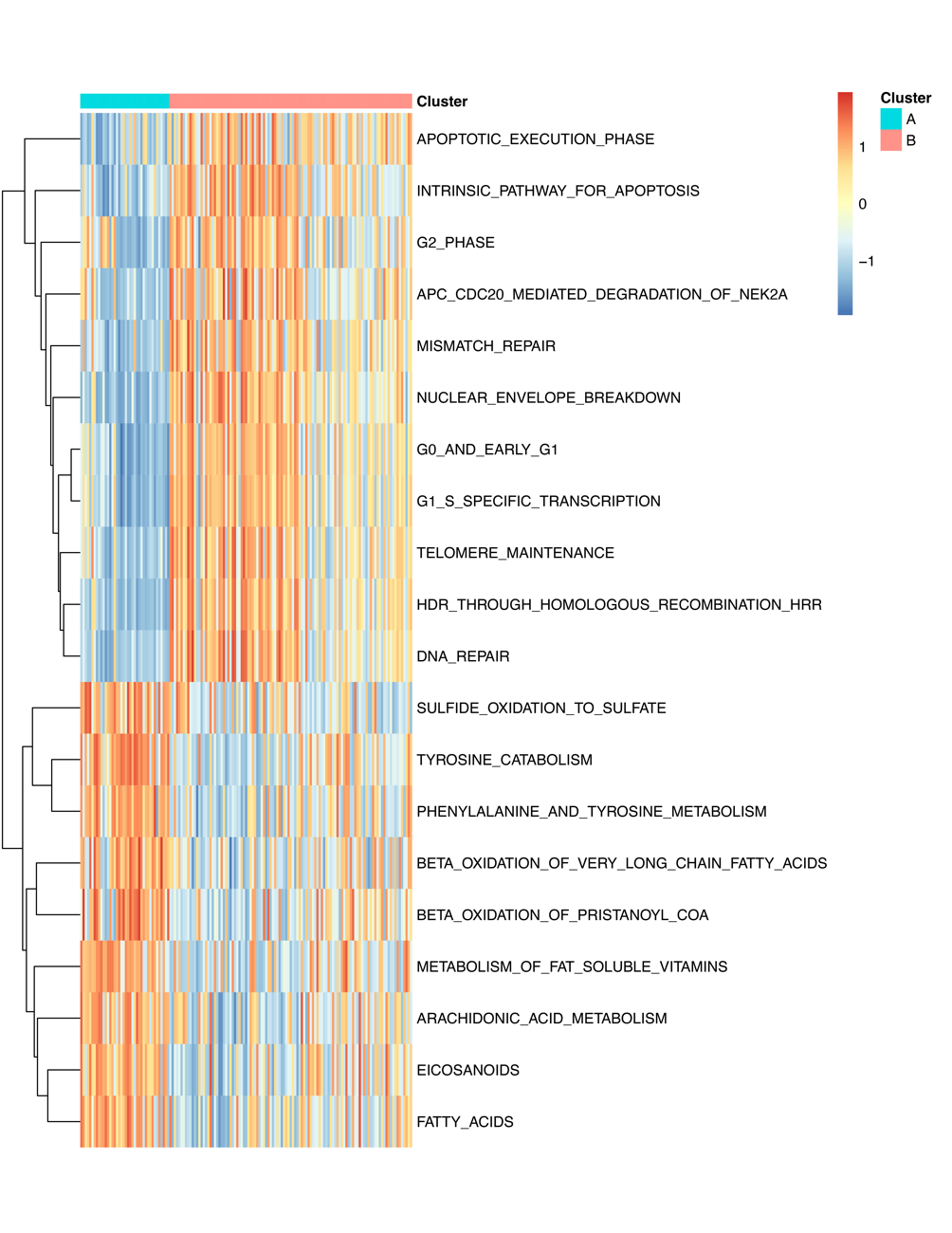


**Fig. S1.** GSVA enrichment analysis showing the activation states of biological pathways in two SUMOylation-related clusters.
