# Supplementary material for: A SUMOylation/immune-related gene signature predicts the prognosis and immunotherapy efficacy of patients with triple-negative breast cancer
Source: PeerJ. 2026 Apr 15;14:e21139. doi: 10.7717/peerj.21139 (PMC13091581; doi:10.7717/peerj.21139)
Supplement: Supplemental Information 2 [file peerj-14-21139-s002.docx]

**Supplementary Figures**

**Article Title**: A SUMOylation/immune-related Gene Signature Predicts the Prognosis and Immunotherapy Efficacy of Patients with Triple-Negative Breast Cancer.

**Fig. S2**


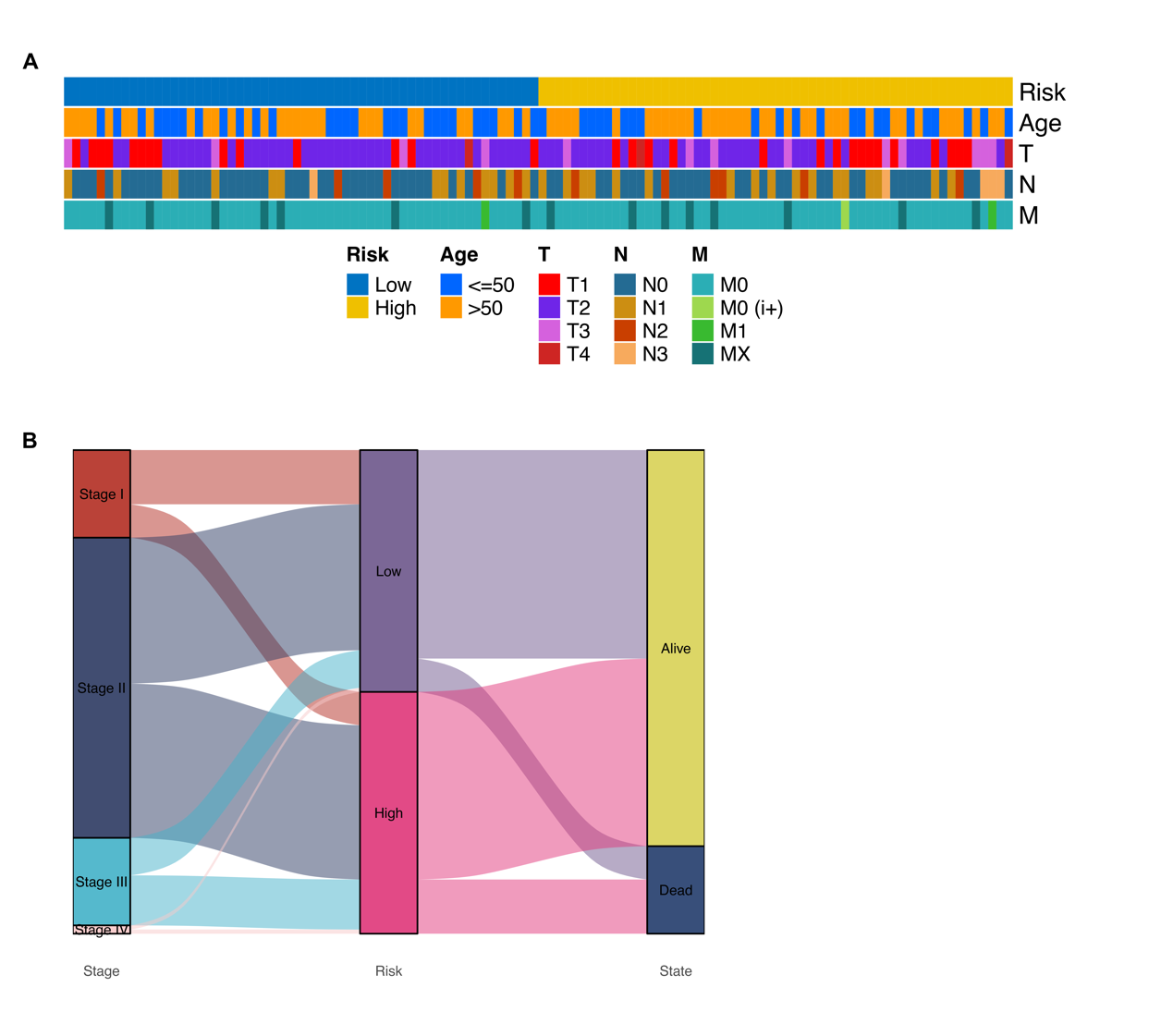


**Fig. S2.** Relationship between clinical characteristics and risk scores. (A) Different clinical characteristics of the high- and low-risk groups. (B) The Sankey diagram showing the connection of clinical stages, the high- and low-risk groups and final survival status.
